# The power of indexers

**DOI:** 10.4102/sajr.v23i1.1827

**Published:** 2019-12-10

**Authors:** Maya Patel

**Affiliations:** 1 National Radiology Services Inc, Johannesburg, South Africa; 2 Department of Radiology, School of Medicine, Faculty of Health Sciences, University of the Witwatersrand, Johannesburg, South Africa

*Academic journalism* is a competitive and complex realm. It is a world of unrelenting struggle for recognition, reputability and acceptance where promoted research is constantly exposed to criticism over quality and relevance to bolster journal metrics.

There are three main aspects of scholarly metrics: impact, speed and reach. Impact factors are based on the number of journal citations, usually over a 2-year period. Speed is related to review turnaround time and time to publication. Reach pertains to global authorship and readership.^1,2^

*Indexing* on the other hand reflects on the journal’s scientific quality. It places the journal on a database, allowing increased visibility, accessibility and readership, and ultimately reputability as a respectable and reliable source in the field of research. The prestige associated with indexing holds relevance for several reasons. Firstly, several academic institutions only recognise indexed publications, particularly for teaching and medical colleagues. Thus, an indexed journal is likely to receive more manuscripts than a non-indexed journal. Secondly, not all indexers are considered equal, despite the availability of several established, well-known databases.^3^ As such, indexing services worldwide have strict inclusion criteria and stringent evaluations prior to acceptance. The difficulty faced by authors and journals is deciding on which indexers to aim for.

Based on the Report on the Evaluation of the 2017 Universities’ Research Output released in March 2019, there has been a steady increase in research productivity across all local universities over the 11-year period evaluated. This is attributed, in part, to institutional strategies and policies that encourage global exposure of local research. The report also documents significant publication growth in South African journals.^4^ These findings affirm a positive research outlook for South Africa and South African journalism, despite the challenges we face.

Supported by the Radiological Society of South Africa, local radiology publishing was first commenced as *Imaging-South Africa/Suid Afrika* in 1990. This was subsequently named the *SA Journal of Radiology (SAJR)* in 1996 and is currently available as an open access digital journal, running its 23rd volume. The *SAJR* is indexed with Scopus (managed by Elsevier); SciELO SA; Web of Science Other Coverage, Emerging Sources Citation Index (ESCI); Directory of Open Access Journals (DOAJ); EBSCO Host; GALE, CENGAGE Learning; Google Scholar; Hinari; ProQuest; African Index Medicus; Norwegian Register for Scientific Journals, Level 1; and has most recently been accepted for inclusion in PubMed Central (maintained by the United States National Library of Medicine). This broad exposure places the journal at an advantage to evolve on multiple platforms. The journal’s new association with the esteemed and established PubMed Central is a significant milestone for carving the future of the *SAJR* in more ways than ever before.

Thank you to all those who have engaged with the *SAJR*, to all those who fostered belief and pride in the journal and to all those who pledged to sustain the journal. Today we are proud to have a radiological journal that continues to reach new heights with a promising future ahead. I stand firm in my goal as the editor to promote the journal and share our research with the world.

‘If we knew what we were doing it would not be called research, would it?’ – Albert Einstein
